# Dark side of a bio-based and biodegradable plastic? Assessment of pathogenic microbes associated with poly(butylene succinate-co-adipate) under ambient and future climates using next-generation sequencing

**DOI:** 10.3389/fpls.2022.966363

**Published:** 2022-10-13

**Authors:** Kantida Juncheed, Benjawan Tanunchai, Sara Fareed Mohamed Wahdan, Katikarn Thongsuk, Martin Schädler, Matthias Noll, Witoon Purahong

**Affiliations:** ^1^Department of Biomedical Sciences and Biomedical Engineering, Faculty of Medicine, Prince of Songkla University, Songkhla, Thailand; ^2^UFZ-Helmholtz Centre for Environmental Research, Department of Soil Ecology, Halle (Saale), Germany; ^3^Bayreuth Center of Ecology and Environmental Research (BayCEER), University of Bayreuth, Bayreuth, Germany; ^4^Department of Botany and Microbiology, Faculty of Science, Suez Canal University, Ismailia, Egypt; ^5^UFZ-Helmholtz Centre for Environmental Research, Department of Community Ecology, Halle (Saale), Germany; ^6^German Centre for Integrative Biodiversity Research (iDiv) Halle-Jena-Leipzig, Leipzig, Germany; ^7^Institute for Bioanalysis, Coburg University of Applied Sciences and Arts, Coburg, Germany

**Keywords:** PBSA, plant pathogens, climate change, interaction effects, human health, opportunistic pathogens

## Abstract

Bio-based and biodegradable plastic mulching films have been proposed to replace the non-biodegradable plastic mulch films to solve plastic pollution problems in agricultural soils. However, the impact of bio-based and biodegradable plastics on plant and human health remains largely unexplored. Here, we aimed to assess the risk under field conditions of a bio-based and biodegradable poly(butylene succinate-co-adipate; PBSA), a widely used mulching film as carrier of potential pathogenic microorganisms (bacteria and fungi) at ambient and future climate conditions. Overall, we affiliated 64 fungal and 11 bacterial operational taxonomic units (OTUs) as pathogens by using Next-Generation Sequencing approach. Our results revealed that PBSA hosted at least 53 plant pathogens, of which 51 were classified as fungi, while the other two were bacteria. Most fungal plant pathogens were able to withstand the anticipated future climate changes. We detected 13 fungal and eight bacterial OTUs, which were classified as opportunistic human pathogens. Only one bacterial OTU (*Enterococcus faecium*) was assigned to a human pathogen. While future climate conditions only significantly impacted on the presence and frequency of detection of few pathogens, incubation time was found to significantly impacted on nine pathogens. This result demonstrates the temporal dynamics of pathogens associated with PBSA. The threats to plant and human health were discussed. We emphasize that the risks to human health are relatively low because we mainly found opportunistic pathogens associated with PBSA and the amount are comparable to the plant debris. However, the risks to plant health may be considered as moderate because many plant pathogens were discovered and/or enriched in PBSA. Furthermore, in soil environments, the pathogenic risk of plastic is highly depending on the surrounding soil pathobiome where plastic is being decomposed.

## Introduction

Nowadays plastics are common in our modern life (Geyer et al., 2017; Purahong et al., 2021). Due to the high demand of plastics during the past decades, the plastic wastes have been widely spread and gathered in the natural environments, involving both aquatic and terrestrial ecosystems (Geyer et al., 2017). Those plastic wastes adversely cause environmental problems, particularly in soil and water pollution, which consequently resulting in plant, animal, and human health impacts (Barnes, 2019). To overcome the accumulation of plastic wastes, the concept of biodegradable polymers was conceived, leading to the commercialization of such plastics (Albertsson and Hakkarainen, 2017). These plastics are considered as eco-friendly plastics since they can be decomposed under various environmental conditions and were less toxic to ecosystems (Haider et al., 2019). Nevertheless, due to limited number of studies, it is still too early to make a generalized conclusion that bio-based and biodegradable plastics can be considered as a sustainable solution solving the plastic pollution problem (Liu et al., 2022; Moshood et al., 2022).

In modern agriculture, mulching films have been widely used to improve water availability and nutrient use efficiency as well as increase temperature in soil, leading to a higher harvesting yield (Touchaleaume et al., 2016; Ngosong et al., 2019). Currently, mulching films made from bio-based and biodegradable plastics such as poly(butylene succinate; PBS), poly(butylene succinate-co-adipate; PBSA) have been extensively replaced conventional plastics due to their mechanical properties closely resemble to polyethylene (PE) and polypropylene (PP; Mizuno et al., 2015). After harvesting season, biodegradable mulch film residues are intentionally disposed in the fields and have been decomposed in agricultural soil (Koitabashi et al., 2012; Serrano-Ruiz et al., 2021). Due to the biodegradability of PBSA, they can degrade under ambient environmental conditions (Emadian et al., 2017) and remain at least 2–3 years in natural soils, hence they can interact with soil microorganisms and environment (Purahong et al., 2021). Not only CO_2_ and H_2_O but also water-soluble monomers, e.g., succinic acid, 1,4-butanediol, and adipic acid are released from PBSA during degradation process (Shah et al., 2013). Those monomers are employed by soil microbes as substrates in the tricarboxylic acid (TCA) or Krebs cycle to produce cellular energy (Lee et al., 2011; Moo-Young, 2011). Furthermore, during decomposition period, PBSA decomposed into macro- and micro-plastics and were colonized by diverse microbial communities, including bacteria and fungi (Emadian et al., 2017; Haider et al., 2019). However, a limited number of adverse effects of PBSA degradation on the soil ecosystem and soil microbial community were identified (Tanunchai et al., 2021). Moreover, most of the reports are based on limited numbers of studies using low-resolution microbiological approaches (Adhikari et al., 2016; Haider et al., 2019). Thus, it is important to apply high-resolution molecular techniques, such as next-generation sequencing (NGS) using Illumina MiSeq sequencing, which offer low detection limit of around 10 cells per mL (Brandt and Albertsen, 2018) to investigate microbial community composition and diversity of using such bio-based and biodegradable plastics in the environment (Crawford et al., 2017).

In a recent study, microorganisms associated in PBSA decomposition process have been investigated using NGS technique (Purahong et al., 2021). This work revealed diverse microbes detected during PBSA decomposition, however, there was no discussion in detail about their function and impact on plant, animal, and human health. These host microorganisms can be categorized into the pathogenic and non-pathogenic microbes (Tanunchai et al., 2021). This might suggest that bio-based and biodegradable plastics may pose dangers to plant and human health, which can be exacerbated by the fact that pathogenic microorganisms can colonize plastics for growth and reproduction (Gan and Zhang, 2019; Tanunchai et al., 2021).

In this study, we re-analyzed the published datasets of bacteria and fungi associated with decomposing PBSA (Purahong et al., 2021) to identify, characterize and evaluate the pathogenic part of microbial PBSA colonizers under field conditions. These bacterial and fungal datasets have been generated using high-resolution molecular techniques based on an experimental case study of PBSA decomposition experiment run over 328 days in an experimental agricultural field at the Global Change Experimental Facility (GCEF; Schädler et al., 2019), which includes a manipulation of the future climate conditions that are expected in central Germany in 50–80 years (in particular reduced summer precipitation by approximately 20%, increased precipitation in spring and autumn by approximately 10% and increased mean temperature by 0.55°C as compared with ambient climate). This enables us to anticipate the temporal dynamics of PBSA-associated pathogens and the negative effects resulting from using of a PBSA in agriculture under anticipated climate conditions. Finally, this study aims to emphasize the negative effects of decomposing bio-based and biodegradable plastics (PBSA) in the agricultural field on plant and human health.

## Materials and methods

### Field experiment, experimental conditions and data analyses

Experimental setup and field conditions have been published in our previous study (Purahong et al., 2021). Briefly, we placed the PBSA films (BioPBS FD92, PTT MCC Biochem company limited, Thailand; in the form of a double-layer thin film 21 cm × 30 cm, thickness 50 μm) on the top of the soil in conventional farming plots (current crop: winter barley, crop rotation: winter rape, winter wheat and winter barley) both in ambient and putative future climate conditions (future climate for Central Germany in the time period 2070–2100, in particular reduced summer precipitation by approximately 20%, increased precipitation in spring and autumn by approximately 10% and increased mean temperature by 0.55°C) of the global change experimental facility (GCEF; Schädler et al., 2019), Bad Lauchstädt, Central Germany (51°22′60″ N, 11°50′60″ E, 118 m a.s.l.) in August 2018 (*ca.* 4 years after climate treatments were effectively manipulated). The PBSA samples were collected after 30 (very early stage), 180 (early stage) and 328 (late stage) days of soil exposure in 5 true replicates, each replicate comes from each individual distinct conventional farming plot (16 m × 24 m) of the GCEF to determine the microbiome of PBSA over 328 days using high throughput sequencing technology. Initial soils without PBSA mulching film in ambient and future putative climate conditions were collected from all experimental plots and used as a control for the microbial community in soil. Soils under PBSA from all experimental plots at 30, 180, and 328 days were also collected to test if PBSA can increase the relative abundances of pathogens within the microbiome of PBSA. The occurrence of potentially pathogenic bacteria and fungi detected in decomposing PBSA under field conditions was investigated.

### Next-generation sequencing and taxonomic assignments

Illumina sequencing was carried out as described in our previous publications (Wahdan et al., 2020, 2021; Purahong et al., 2021). Briefly, PBSA samples were subsampled and removed loosely adherent soil particles by vortexing in sterile phosphate-buffered saline (0.01 M) for 5 min. PBSA samples were then submerged and shaken vigorously in 45 mL sterile Tween (0.1%) and this step was repeated 3 times. The samples were then washed 7 times using sterile water. Microbial biomass attached firmly with PBSA sample was then subjected to DNA extraction using DNeasy PowerSoil Kit (Qiagen, Hilden, Germany) with the aid of a Precellys 24 tissue homogenizer (Bertin Instruments, Montigny-le-Bretonneux, France) according to manufacturers’ instructions. DNA samples were used for Illumina sequencing. For bacterial and archaeal amplicon library, the 16S rRNA gene V4 region was amplified using the universal bacteria/archaea primer pair 515F (5′-GTGCCAGCMGCCGCGGTAA-3′) and 806R (5′ -GGACTACHVGGGTWTCTAAT-3′; Caporaso et al., 2011). For the fungal amplicon libraries, the fungal internal transcribed spacer 2 (ITS2) gene was amplified using the fungal primer pair fITS7 (5′-GTGARTCATCGAATCTTTG-3′; Ihrmark et al., 2012) and ITS4 primer (5′-TCCTCCGCTTATTGATATGC-3′; White et al., 1990). Amplifications were performed in 20 μl reactions with 5x HOT FIREPol Blend Master Mix (Solis BioDyne, Tartu, Estonia). The products from 3 technical replicates were then pooled equimolar. Paired-end sequencing of 2 × 300 bp of this pool was performed using a MiSeq Reagent kit v3 on an Illumina MiSeq system (Illumina Inc., San Diego, CA, United States) at the Department of Soil Ecology, Helmholtz Centre for Environmental Research. We screened for high-quality data as described in our previous work (Weißbecker et al., 2020; Purahong et al., 2021). SILVA database v132 for prokaryote 16S (Yilmaz et al., 2014) and UNITE database (version unite.v7; Kõljalg et al., 2013; Nilsson et al., 2019) were used for bacterial and fungal taxonomic assignment using 97% threshold. Sequences which potentially originate from sequencing errors (chimeras sequences, rare taxa, especially the sequences detected once) were removed from our datasets. From the minimum sequencing reads, we obtained 31,141 for bacteria and archaea, and 26,056 for fungi. Rarefaction curves were saturated for all samples. Ecological functions were affiliated for each OTU using FAPROTAX for bacteria and archaea (Louca et al., 2016), and FUNGuild for fungi (Nguyen et al., 2016). Plant and human pathogens were extracted and separated from other functional groups and used in this study (Supplementary Tables S1–S3). We checked the taxonomic annotation of all pathogenic bacterial and fungal OTUs used in this study by BLAST search against NCBI (bacteria, at 100% threshold) and the current version of UNITE (version: 8.2; 2020-01-15, fungi, at 99% threshold). Pathogens are classified as plant pathogens [including, cereal crops, vegetables, fruits, ornamental plants and trees; the host plants are identified according to database of UNITE (Nilsson et al., 2019)] and opportunistic human pathogens. We also manually checked all identified pathogenic bacteria and fungi using other references in ISI database. In this study, we assessed risks for plants and humans by comparing plant and human pathogens in wheat straw with PBSA, which decomposed in vicinity of PBSA in the field (Wahdan et al., 2020). The raw 16S and ITS rDNA sequences were deposited in the National Center for Biotechnology Information (NCBI) Sequence Read Archive (SRA) under study accession number PRJNA595487.

### Statistical analysis

The effects of climate, incubation time and their interaction on the relative abundances (frequency of detection) of individual pathogens were assessed by time-series analysis (repeated ANOVA) using SPSS as the data sets vary over time. With this test, climate was used as a between-subject factor and time was used as within-plot factor. All data sets were tested for normality and equality of variances using the Shapiro–Wilk test and Levene’s test, respectively. Data were log 10 transformed when necessary.

## Results

### General overview of pathogens detected in decomposing PBSA

**Figure 1:**
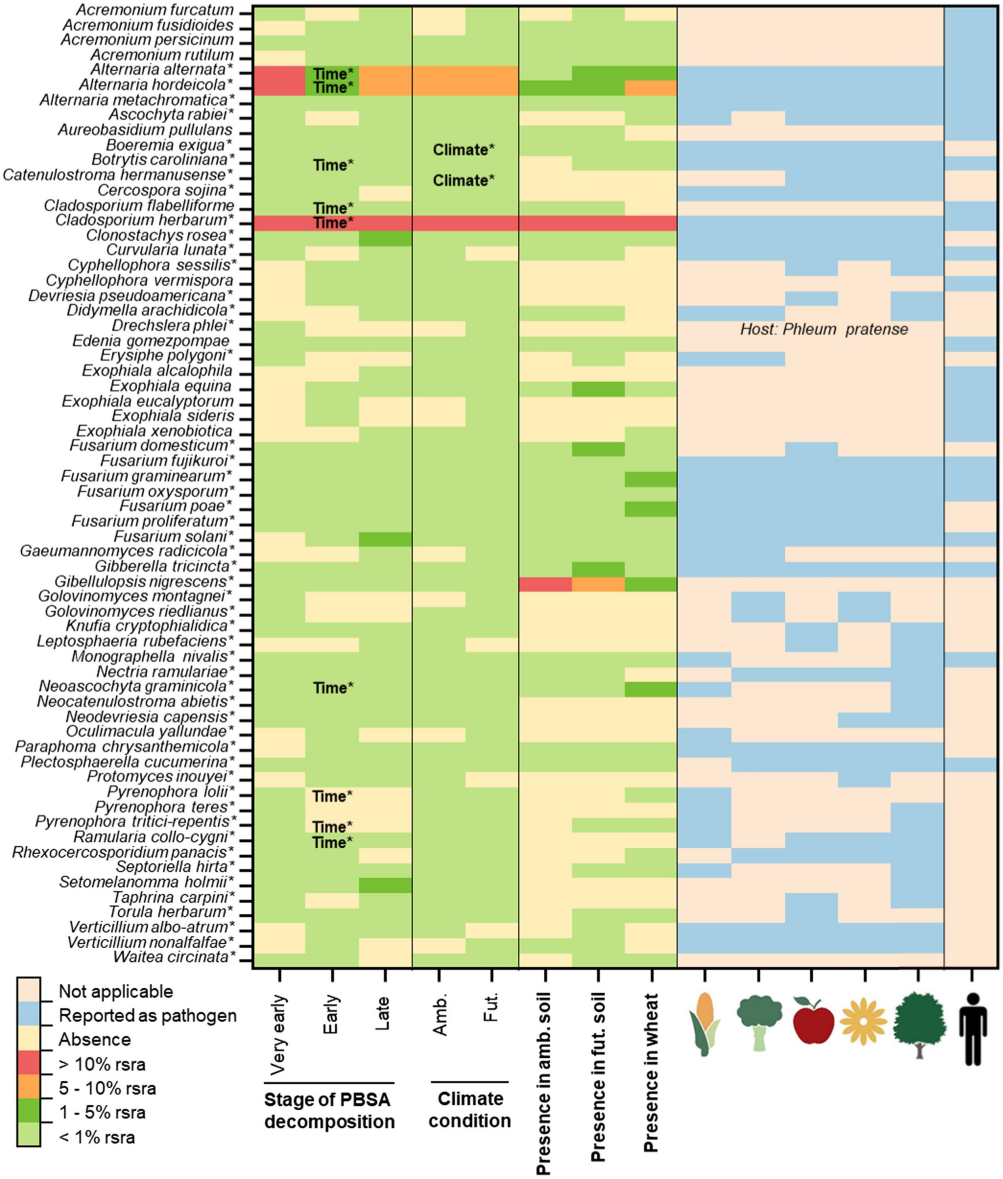
The occurrence of potentially pathogenic fungi detected in decomposing PBSA films (at very early, early and late stages of decomposition) in soils of ambient (amb.) and future putative (fut.) climate conditions using next generation sequencing (NGS). Their presence in early decomposing wheat straw is also shown. Pathogens are separated as plant pathogens [including, pathogens of different plant functional groups: cereal crops (corn symbol), vegetables (vegetable symbol), fruits (apple symbol), ornamental plants (flower symbol) and trees (tree symbol); the host plants are associated with the UNITE Species Hypothesis (SH) of each plant pathogen identified according to database of UNITE (Nilsson et al., 2019)] and opportunistic human pathogens (human symbol; Supplementary Table S1). The symbol (*) indicates the confirmation of status “plant pathogen” accoding to relevant references (Supplementary Table S1). Time^*^ and climate^*^ indicate significant effects accoding to repeated measure ANOVA (*p* < 0.05) (Supplementary Table S6). The abbreviation “rsra” refers to relative sequence read abundances.

**Figure 2:**
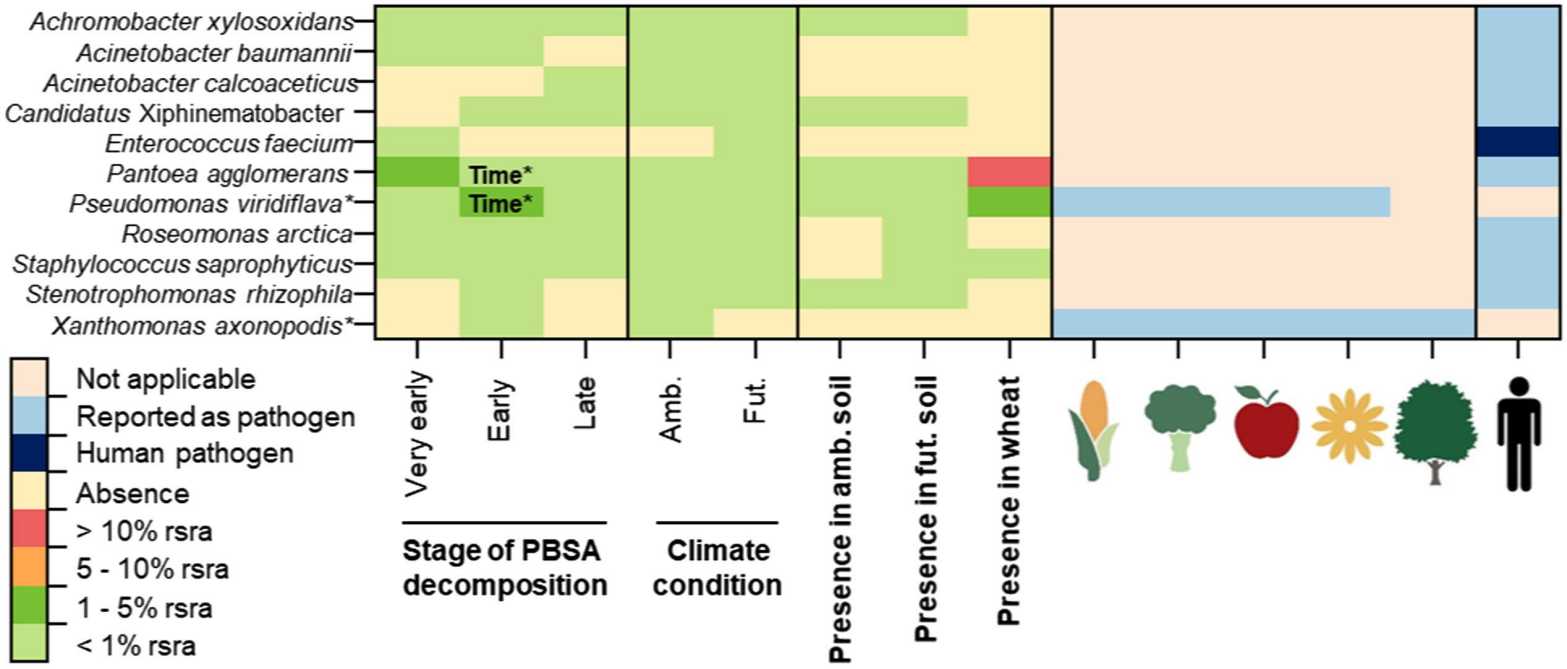
The occurrence of potentially pathogenic bacteria detected in decomposing PBSA films (at very early, early and late stages of decomposition) in soils of ambient (amb.) and putative future (fut.) climate conditions using next generation sequencing (NGS). Their presence in early decomposing wheat straw is also shown. Pathogens are separated as plant pathogens (including, pathogens of different plant functional groups: cereal crops, vegetables, fruits, ornamental plants and trees), opportunistic human pathogens and human pathogens identified according to relevant references (Supplementary Table S1). Symbols of each plant and human pathogen are introduced in Figure 1. Time^*^ indicates significant effect accoding to repeated measure ANOVA (*p* < 0.05; Supplementary Table S7). The abbreviation “rsra” refers to relative sequence read abundances.

Overall, we detected 64 fungal and 11 bacterial OTUs as pathogens (Figures 1, 2). There were 51 fungal and two bacterial OTUs classified as plant pathogens. Thirteen fungal OTUs and eight bacterial OTUs were classified as opportunistic human pathogens (Figures 1, 2). Only *Enterococcus faecium* was recognized as a human pathogen. Additionally, some pathogens were classified as plant pathogens as well as opportunistic human pathogens (Figures 1, 2). The majority of these bacterial (8/11 OTUs) and fungal (44/64 OTUs) pathogens were also detcted in control soils without mulching films (Supplementary Tables S4, S5). Few fungal pathogens associated with PBSA, especially those dominant ones (i.e., *Alternaria alternata*, *Alternaria hordeicola*, and *Cladosporium herbarum*) were more frequently detected in soils after PBSA addition (Supplementary Table S4).

In detail, our field study showed that PBSA hosted 21 opportunistic human pathogens, with fungi being the dominant pathogens in our data set (13/21 opportunistic human pathogenic OTUs; Figures 1, 2; Supplementary Tables S1–S3). These fungi included *Acremonium furcatum*, *Acremonium fusidioides*, *Acremonium persicinum*, *Acremonium rutilum*, *Aureobasidium pullulans*, *Cladosporium flabelliforme*, *Cyphellophora vermispora*, *Edenia gomezpompae*, *Exophiala alcalophila*, *Exophiala equina*, *Exophiala eucalyptorum*, *Exophiala sideris*, and *Exophiala xenobiotica*. Eight bacterial OTUs including *Achromobacter xylosoxidans*, *Acinetobacter baumannii*, *Acinetobacter calcoaceticus*, *Candidatus* Xiphinematobacter, *Pantoea agglomerans*, *Roseomonas arctica*, *Staphylococcus saprophyticus*, and *Stenotrophomonas rhizophila* were classified as opportunistic human pathogens.

For plant pathogens, our field study showed that PBSA hosted 53 plant pathogens, with fungi being the predominant plant pathogens in our data set (51/53 plant pathogen OTUs; Figure 1; Supplementary Tables S1–S3). The examples of important fungal plant pathogens detected in PBSA were *A. alternata*, *A. hordeicola*, *C. herbarum*, *Clonostachys rosea*, *Fusarium solani*, *Gibberella tricincta*, *Gibellulopsis nigrescens*, *Pyrenophora lolii*, *Septoriella hirta*, and *Setomelanomma holmii*. There were only two bacterial pathogenic species (*Pseudomonas viridiflava* and *Xanthomonas axonopodis*) out of 53 plant pathogens detected. Interestingly, *P. agglomerans* was classified as both plant and opportunistic human pathogen (Figure 2; Supplementary Table S3).

### Stages of PBSA degradation (incubation time) and future climate on detection of pathogens

The microbial communities during PBSA decomposition at different time points (30, 180, and 328 days) were investigated. We found that some pathogens exhibit stable relative abundance over time whereas some pathogens showed significant temporal dynamics. We found that incubation time significantly influenced the detection of nine pathogenic fungal OTUs [*A. alternata* (*F* = 25.46, *p* < 0.001), *A. hordeicola* (*F* = 15.03, *p* = 0.003), *Botrytis caroliniana* (*F* = 8.20, *p* = 0.004), *C. flabelliforme* (*F* = 11.65, *p* = 0.005), *C. herbarum* (*F* = 175.83, *p* = 0.000), *Neoascochyta graminicola* (*F* = 17.78, *p* = 0.003), *Pyrenophora lolii* (*F* = 5.42, *p* = 0.048), *Pyrenophora tritici-repentis* (*F* = 8.31, *p* = 0.020), and *Ramularia collo-cygni* (*F* = 6.02, *p* = 0.031)] and two pathogenic bacterial OTUs [*P. agglomerans* (*F* = 13.11, *p* = 0.007) and *P. viridiflava* (*F* = 11.71, *p* = 0.009; Figures 1, 2; Supplementary Tables S6, S7)].

We observed three patterns of microbial relative abundances among those pathogens. First, the relative abundances of *A. alternata*, *A. hordeicola*, *B. caroliniana*, *C. herbarum*, *N. graminicola*, *P. lolii*, and *P. tritici-repentis* were found to be high at a very early stage (30 days of PBSA soil exposure) and decreased as PBSA decomposition progressed. Second, the relative abundances of *C. flabelliforme* and *R. collo-cygni* were low at a very early stage (30 days) of PBSA decomposition but increased as the decomposition progressed. Finally, the relative abundances of *P. agglomerans* and *P. viridiflava* were enriched in the early stage (180 days) of PBSA decomposition, and their relative abundances decreased at 328 days.

This experiment examined not only the impact of incubation time but also the effect of future putative climate conditions compared to ambient climate conditions and interaction between incubation time and respective climate conditions. We found that future putative climate (reduction of precipitation and increasing of mean temperature compared to ambient climate) impacts on two fungal pathogens [*Boeremia exigua* (*F* = 8.73, *p* = 0.018) and *Catenulostroma hermanusense* (*F* = 6.61, *p* = 0.033; Supplementary Table S6)]. We revealed that *B. exigua* was more abundant in the ambient climate than in the future putative climate conditions, but *C. hermanusense* was more prevalent in the future climate conditions. The interaction between incubation time and future climate conditions was not significant (*p* > 0.05) for any pathogens (Supplementary Tables S6, S7).

## Discussion

### Effect of incubation time and putative future climate changes on detection of pathogens associated with decomposing PBSA

Climate change by raising temperatures and changing precipitation patterns can alter soil microbial communities (Wahdan et al., 2020; Kostin et al., 2021), but its effects on microbial colonization of plastic are rarely studied. As a result, we know relatively little about the whole microbial communities and their dynamics associated with bio-based and biodegradable plastics, as well as their potential interactions in putative future climate condition scenario (Šerá et al., 2020; Purahong et al., 2021). Although NGS approach can be used to analyze the whole microbial community composition as well as their specific functions (i.e., pathogens, saprotroph, symbiotrophs, etc.), it has been rarely employed to evaluate the microbiome of biodegradable plastics under soil conditions (Kamiya et al., 2007; Shokralla et al., 2012; Purahong et al., 2021). Pathogenic microbes in this study were not only present by chance on decomposing PBSA, but there were many pathogens, especially the fungal ones (such as *A. alternata*, *F. solani,* and *C. herbarum*), which are known to be efficient PBSA decomposers as they can produce particular enzymes such as lipases and esterases that catalyze the breakdown of the PBSA polymer chain (Emadian et al., 2017; Satti and Shah, 2020). In this study, we showed that the incubation time has significant effect on nine plant pathogenic fungal and two plant pathogenic bacterial OTUs. For this, it is possible to infer that plant pathogens exhibit temporal dynamics on decomposing PBSA. This is due to the fact that there is a shift in the quality of the PBSA (i.e., changes in molar mass, surface structure, and area) as well as seasonal changes, both of which have the potential to change the relative abundances and interaction patterns of microbial members within the microbial communities (Purahong et al., 2021). Thus, disease spread from PBSA-associated pathogens may depend on the load of such pathogens, which can be affected by decomposition stage of PBSA.

Overall, we showed that the majority of fungal plant pathogens were able to withstand the future putative climate conditions. However, we also found that future putative climate conditions had a significant effect on two plant pathogenic fungal OTUs (*B. exigua* and *C. hermanusense*; Figure 1). Specifically, *B. exigua* were more frequently detected in ambient than in future putative climate conditions, but *C. hermanusense* were more frequently detected in the future putative climate. *B. exigua* is capable to cause field pea black spot disease in *Pisum sativum* and *Glycine max* (Li et al., 2012; Schaffrath et al., 2021). This disease typically affects the majority of plants and pea fields during the early winter/spring months (Li et al., 2012). This supports our finding that future putative climate conditions with a higher average temperature would reduce the prevalence of *B. exigua*, as this microbe adapted to lower temperature environments. In contrast, future putative climate conditions increased the sequence read abundances of *C. hermanusense* and *C. germanicum* (syn. *Neocatenulostroma germanicum*), which can cause needle blight symptoms on *Pinus mugo*, *Pinus nigra* subsp. *pallasiania*, and *Pinus sylvestris* (Markovskaja et al., 2016). Genus *Catenulostroma* belong to a group of black, melanized, extreme-tolerant capnodialean micro fungi. These fungi can survive in extremely cold, hot, dry or salty environments (Dadachova et al., 2007; Gorbushina et al., 2008; Selbmann et al., 2013). This suggests that members of the genus *Catenulostroma* may prefer to flourish in harsh conditions, such as the future putative climate conditions. The interaction effects between time and climate, on the other hand, were not significant in any fungal or bacterial OTUs. Thus, the results can be concluded that future putative climate conditions consistently impact on the detection of pathogens associated with decomposing PBSA over time. Nevertheless, exposure time showed stronger effect than future putative climate conditions. Our previous work revealed that a succession of microbial communities played a key role in detection of plant pathogenic fungi associated with decomposing wheat straw (Wahdan et al., 2020).

### Risk of decomposing PBSA to plants and agriculture

In this study, we used wheat straw as an alternative substrate to PBSA, which decomposed in the field as the plant litter residues. These plant residues can act as a bridge between plants and the soil as they decay during one or more cropping seasons (Wahdan et al., 2020). Plant residues can be considered as an important source of plant pathogen propagules. These residue-borne pathogens may result in severe agricultural yield loss and mycotoxin contaminations (Kerdraon et al., 2019). To reasonably estimate the risks of bio-based and biodegradable plastics in hosting fungal and bacterial plant pathogens, we compared the presence and the frequency of fungal and bacterial plant pathogens during decomposition of PBSA and decomposing wheat straw (known as an important source of pathogen inoculum when returned to soil), adding to the same plots in the vicinity of PBSA (Wahdan et al., 2020). We found that many fungal and bacterial pathogens were enriched in wheat straw (i.e., fungi: *A. alternata*, *A. hordeicola*, *C. herbarum*, *Fusarium graminearum*, *Fusarium poae*, *G. nigrescens*, *Neoascochyta graminicola*; bacteria: *P. agglomerans*, *P. viridiflava*, and *S. saprophyticus*; Figures 1, 2; Supplementary Tables S1–S3). We found that the majority of these fungal and bacterial plant pathogens were also frequently detected in PBSA, however the decomposition of wheat straw harbor higher richness of plant pathogens as compared with PBSA.

The ability of plant pathogens to colonize and degrade PBSA is possible and can be seen as important risk to nearby plants. Most fungal plant pathogens detected in PBSA were linked to at least two plant functional groups, with nearly 50% linked to all plant functional groups, including cereal crops, vegetables, fruits, ornamental plants, and trees (Figure 1; Supplementary Tables S1, S2). Because the majority of plant pathogens were detected at all sampling time points (including the very early (30 days), early (180 days), and late (328 days) stages of PBSA degradation), thus they may have enough time to infect nearby plants during the growing seasons. The majority of fungal plant pathogenic species were also able to resist forecasted future climate conditions. These microorganisms (i.e., *A. alternata*, *A. hordeicola*, *C. herbarum*, *C. rosea*, *Devriesia pseudoamericana*, *E. equina*, *Fusarium oxysporum*, *Fusarium proliferatum*, *F. solani*, *G. tricincta*, *G. nigrescens*, and *Paraphoma chrysanthemicola*) can metabolize PBSA due to their genetic capacity to synthesize essential enzymes such as lipases, esterases or catalase (Emadian et al., 2017; Satti and Shah, 2020). Some of these fungi have been identified to be effective lipase producers and/or PBS/PBSA degraders (Emadian et al., 2017; Purahong et al., 2021). *F. solani* is one of the most common plant pathogens, infecting a wide range of economically important plant species and plant functional groups (Coleman, 2016), and causing diseases such as garden pea root rot (*Pisum sativum*; Coleman, 2016), soybean sudden death syndrome (Coleman, 2016), and foot rot in potatoes (*Solanum tuberosum*) and tomatoes (*Lycopersicon esculentum*; Romberg and Davis, 2007). Thus, *F. solani* can cause significant economic losses. Additionally, Scheid et al. established a model system that revealed a relationship between mung bean (*Vigna radiata L.*) and a plant pathogen that was enriched by PBSA amended soil (*F. solani*; Scheid et al., 2022). Based on such short-term experiments, they found that PBSA alone may not negatively impact on biomass and health of seedlings, however interaction between N fertilizer and PBSA can strongly negatively affect plant health (Scheid et al., 2022). Taking all results together, leaving mulching films (PBSA) in agricultural fields with N fertilization after harvesting may result in decreased agricultural productivity, as *F. solani* and other plant pathogens may have time to grow and giving them a good starting position to interact with plants even under striking conditions. Moreover, PBSA films may also provide habitat and nutrient sources for some plant pathogens of neighboring plants and its microbial communities. Wind, water, and animal vectors can spread plant pathogens from the neighboring plants to the crops and cause losses to the yield.

Apart from *F. solani*, numerous of other important crop pathogens were detected and/or enriched in PBSA, including *G. nigrescens* (which causes vein-delimited yellowing of leaves and light-brown vascular discoloration of petioles in sugar beet; Zhou et al., 2017), *C. herbarum* (which causes strawberry blossom blight and wheat leaf spots; Perelló et al., 2003; Nam et al., 2015), and *A. alternata* (which causes leaf blight disease in onions and leaf spot disease in tomatoes; Ramjegathesh and Ebenezar, 2012; El-Gazzar and Ismail, 2020). Apart from its role as a plant pathogen infecting faba bean (*Vicia faba*; Afshari and Hemmati, 2017), the fungus *C. rosea* is known to act as a biological control agent against grey mold disease caused by *Botrytis cinerea* in tomato (Mouekouba et al., 2014) and strawberry (Cota et al., 2009). Bacterial plant pathogens *P. agglomerans* and *P. viridiflava* were enriched in the very early and early stages of PBSA degradation. These bacterial plant pathogens are reported to infect a broad range of host plants (Sarris et al., 2012; Mhedbi-Hajri et al., 2013).

### Risk of decomposing PBSA to animal and human health

While the risk of PBSA to plants is moderate and may depend on incubation time, fertilization strategies, climate conditions and interaction with other environmental factors, the risk to human health is considered to be as low as compared to the amendment of wheat straw. In our field experiment, we only detected some opportunistic human pathogens (8 bacterial pathogen species and 13 fungal pathogen species) and one human pathogen that participated in the colonization of PBSA. *E. faecium* was the only bacterial human pathogen detected in this study, which is classified as a safety level WHO priority 2 pathogen (Asokan et al., 2019), as it can cause severe infections of the urinary tract and bloodstream (bacteremia), as well as infections of the abdomen and endocarditis (Selleck et al., 2019), and is resistant to most antibiotics (Miller et al., 2014). However, it was detected at a very low relative sequence read abundance and only in the very early stages of PBSA decomposition under future putative climate conditions. This may raise the low to moderate possibility that future climate conditions may result in increasing of infections caused by pathogens associated with bio-based and biodegradable polymers. Nevertheless, this is probably unlikely situation because the opportunistic bacterial pathogens discovered in our field study were present at very low to extremely low frequency (Supplementary Table S2). *P. agglomerans* was found in the early stages of PBSA decomposition, though its relative abundance decreased significantly between the early and late stages. Human pathogens found in PBSA were also found in wheat straw and agricultural soil (Figures 1, 2). Human pathogens are well-known to be part in soil microbial communities and for their ability to colonize a variety of substrates, including plant materials and bio-based and biodegradable polymers. Because most of the pathogens discovered in PBSA were opportunistic human diseases, the presence of PBSA is considered to pose a low risk to human health.

### The dark side of a bio-based and biodegradable plastic in environmental health

Development of bio-based and biodegradable plastics might be an effective step toward reducing global plastic pollutions (Wang et al., 2021) as they can save non-renewable resources in the production process and reduce CO_2_ and greenhouse gases (GHGs) emission (Jiang et al., 2015; Haider et al., 2019). Nonetheless, these plastics would still influence the environment throughout their usage. Thus, it is necessary to estimate their impact on environmental health. In this study, we showed in-depth results from PBSA. However, there are other polyester-based plastics, which are considered as promising biodegradable plastics for mulching films, such as polybutylene succinate (PBS), polyhydroxyalkanoate (PHA), and polybutylene adipate terephthalate (PBAT) which should also be tested in the future (Gigli et al., 2012; Albertsson and Hakkarainen, 2017; Satti and Shah, 2020). The biodegradable films contribute significantly to plastic pollution in terrestrial ecosystems (Liu et al., 2014), since they are often tilled into the soil and left to decompose after harvesting (Koitabashi et al., 2012; Serrano-Ruiz et al., 2021). Indeed, it is anticipated that soil will serve as a substantial sink for plastic waste in agriculture beside landfills (Horton et al., 2017). Microbes present in soil have been demonstrated to be vital for biodegradable plastics degradation (Zumstein et al., 2018). Recent research established that soil microorganisms, particularly filamentous fungi and bacteria, are capable of degrading a common biodegradable plastic (PBAT) used in agriculture and utilizing the carbon of each monomer unit for growth and metabolism (Zumstein et al., 2018). Meanwhile, the breakdown of bio-based and biodegradable plastics, plastic pieces (macro-plastics) and decomposed chemical compounds such as plasticizers, dyes, and other chemical compounds may be released, causing chemical pollution of the soil, alteration of the soil’s physical structure, and biological contamination with pathogens (Briassoulis and Giannoulis, 2018; Wan et al., 2019).

### Pathobiome of the surrounding environment play important role in determining the risk of plastics

In this study, we analyzed the control soils to adequately assess the risk related to soil microbiota itself. Our results demonstrate that the pathogenic risk of plastic is also depending on the soils where plastic is being decomposed. The traditional petroleum-based plastic and bio-based and biodegradable plastics are both associated and able to be colonized by plant and human pathogens (Supplementary Table S8; Gan and Zhang, 2019). A previous study based on high-resolution chemical methods also found that metabolites emitted from bio-based and biodegradable plastics are not safer than traditional petroleum-based plastics (Zimmermann et al., 2020). In general, plastics can act as a medium and as a substrate for colonization of pathogens, thus pathobiome of the surrounding environment can play important role in determining the environmental risk of plastic pollutions.

## Conclusion

This work revealed that many plant pathogenic microbes can be detected and/or enriched in PBSA under field conditions, thus the risk for plant health can be considered as moderate. We therefore need to find other options to reduce the negative effects of bio-based and biodegradable plastics that might influence agricultural production practices. For this reason, waste management and recycling of those eco-friendly plastics might be a helpful alternative to leaving bio-based and biodegradable plastics to decompose in the field. In contrast, the risk to human health is relatively low because we mainly found opportunistic pathogens associated with PBSA and the amount are comparable to the plant debris. However, the chance of human infections caused by plant pathogens is an unknown issue that raises critical questions considering the tendency of such infections to occur in healthy individuals. In our opinion, there are still limited number of studies looking at the risk assessment of bio-based and biodegradable plastics to plants and human health, so we should be cautious in our decisions regarding bio-based and biodegradable plastics. Furthermore, in soil environments the pathogenic risk of plastics is also depending on the surrounding soil pathobiome where plastic is being decomposed.

## Data availability statement

The datasets presented in this study can be found in online repositories. The names of the repository/repositories and accession number(s) can be found at: www.ncbi.nlm.nih.gov/bioproject/PRJNA595487.

## Author contributions

WP conceptualized and designed the research and provided reagents and laboratory equipment. BT, WP, and SW led the laboratory experimental setup. MS maintained the GCEF. BT, WP, and KJ evaluated the samples and metadata, led the microbial taxonomy and data processing, and wrote the manuscript. MN and KT edited the manuscript and contributed to the figures. BT, WP, KJ, and SW led the DNA analysis. KJ led statistical analysis. All authors contributed to the article and approved the submitted version.

## Funding

This work has been partially funded by the internal research budget of WP to the Department of Soil Ecology, UFZ-Helmholtz Centre for Environmental Research.

## Conflict of interest

The authors declare that the research was conducted in the absence of any commercial or financial relationships that could be construed as a potential conflict of interest.

## Publisher’s note

All claims expressed in this article are solely those of the authors and do not necessarily represent those of their affiliated organizations, or those of the publisher, the editors and the reviewers. Any product that may be evaluated in this article, or claim that may be made by its manufacturer, is not guaranteed or endorsed by the publisher.
