# Supplementary material for: Dark side of a bio-based and biodegradable plastic? Assessment of pathogenic microbes associated with poly(butylene succinate-co-adipate) under ambient and future climates using next-generation sequencing
Source: Front Plant Sci. 2022 Oct 13;13:966363. doi: 10.3389/fpls.2022.966363 (PMC9610124; doi:10.3389/fpls.2022.966363)
Supplement: Supplementary file 1 [file Data_Sheet_1.pdf]

Supplementary material

Dark side of a bio-based and biodegradable plastic? Assessment of pathogenic microbes associated with poly(butylene succinate-co-adipate) under ambient and future climates using next generation sequencing

Kantida Juncheed [1,2†], Benjawan Tanunchai [2,3†], Sara Fareed Mohamed Wahdan [2,4], Katikarn Thongsuk[2], Martin Schädler [5,6], Matthias Noll [3,7], Witoon Purahong [2,¶,*]

[1]Department of Biomedical Sciences and Biomedical Engineering, Faculty of Medicine, Prince of Songkla University, Songkhla, 90110 Thailand

[2]UFZ-Helmholtz Centre for Environmental Research, Department of Soil Ecology, Theodor-Lieser-Str. 4, 06120 Halle (Saale), Germany

[3]Bayreuth Center of Ecology and Environmental Research (BayCEER), University of Bayreuth, 95440, Bayreuth,

Germany

[4]Department of Botany and Microbiology, Faculty of Science, Suez Canal University, 41522 Ismailia, Egypt

[5]UFZ-Helmholtz Centre for Environmental Research, Department of Community Ecology, Theodor-Lieser-Str. 4, 06120 Halle (Saale), Germany

[6]German Centre for Integrative Biodiversity Research (iDiv) Halle-Jena-Leipzig, Deutscher Platz 5e, 04103 Leipzig, Germany

[7]Institute for Bioanalysis, Coburg University of Applied Sciences and Arts, 96450 Coburg, Germany

[†] These authors contributed equally to this work

[¶] Senior Authors

[*] Correspondence:

Witoon Purahong

witoon.purahong@ufz.de

Table S1. List of the host plants associated with the UNITE Species Hypothesis (SH) of each plant pathogen identified according to database of UNITE (Nilsson et al., 2019)*.

UNITE Species Hypothesis	Note	SH codes	% Matched	Dectected location	Plant host/Interacting taxa
Acremonium furcatum	Synonym, *Gibellulopsis piscis*	SH1234027.08FU	100	Worldwide	*Brassica napus (7); Dittrichia viscosa (3); Beta vulgaris (3); Cicer arietinum (2); Elymus farctus (1); Oryza granulata (1); Phoenix canariensis (1); Washingtonia robusta (1); Populus simonii (1); Aralia cordata (1); Vitis vinifera (1); Helianthus annuus (1); Solanum tuberosum (1); Zea mays (1); Diabrotica virgifera subsp. virgifera (1); Cymodocea nodosa (1); Microthlaspi perfoliatum (1); Posidonia oceanica (1); Fagus sylvatica (1);*
Acremonium fusidioides	This name is not in taxonomy, UNITE = *Acremonium alternatum*	SH1182855.08FU	87	Europe	-
Acremonium persicinum	Correct	SH1148339.08FU	100	Africa America Asia Australia Europe	*Poaceae (4); Eucalyptus (1); Vitis vinifera (1); Homo sapiens (1); Cecropia insignis (1); Artemisia annua (1); Anthozoa (1); Microthlaspi perfoliatum (1); Codium fragile (1);*
Aureobasidium pullulans	Correct	SH1149661.08FU	100	Worldwide	*Fagus (7); Vitis (6); Populus tremula (4); Vitis vinifera (4); Festuca rubra (3); Zea mays (3); Ferula (3); Populus nigra (3); Malus domestica (3); Proteaceae (3); Alnus glutinosa (2); Humulus lupulus (2); Pinus halepensis (2); Pinus radiata (2); Calamagrostis purpurea (2); Festuca vivipara (2); Fraxinus excelsior (2); Calluna vulgaris (2); Quercus (2); Arabidopsis thaliana (1); Populus angustifolia (1); Triticum aestivum (1); Prunus avium (1); Alnus incana (1); Quercus cerris (1); Quercus robur (1); Salix nigricans (1); Phleum pratense (1); Salix appendiculata (1); Taxus chinensis var. mairei (1); Populus trichocarpa (1); Castanea sativa (1); Pinus sylvestris (1); Acer saccharum (1); Holcus lanatus (1); Artemisia vulgaris (1); Malus sylvestris (1); Convallaria majalis (1); Brassica napus (1); Homo sapiens (1); Picea glauca (1); Diabrotica virgifera (1); Eucalyptus (1); Plantae (1); Viscum album subsp. austriacum (1); Quercus rubra (1); Prunus persica (1); Populus euphratica (1); Magnolia grandiflora (1); Solanum tuberosum (1); Arbutus unedo (1);*
Cladosporium flabelliforme	This name is not in taxonomy, UNITE name = *Mycosphaerella tassiana*	SH1190878.08FU	98	Worldwide	*Zea mays (20); Juniperus deppeana (10); Coffea arabica (6); Picea abies (6); Hevea brasiliensis (4); Phragmites australis (4); Vitis (4); Cecropia insignis (3); Acropora formosa (2); Rhododendron fortunei (2); Quercus (2); Pinus monticola (2); Yucca glauca (2); Carica papaya (2); Vitis vinifera (2); Helianthus annuus (1); Epacris microphylla (1); Glycine max (1); Fagus sylvatica (1); Eucalyptus grandis (1); Saccharum officinarum (1); Poaceae (1); Ostrinia nubilalis (1); Rosa (1); Quercus nigra (1); Trifolium repens (1); Medicago polymorpha (1); Angiospermae (1); Platycladus orientalis (1); Pyrus communis (1); Calluna vulgaris (1); Ginkgo biloba (1); Silene maritima (1); Pinus ponderosa (1); Taxus globosa (1); Nothofagus cunninghamii (1); Quercus petraea (1); Sorghum vulgare (1); Grevillea robusta (1); Juniperus virginiana (1); Cucumis sativus (1); Paphiopedilum bellatulum (1); Cortaderia (1); Eucalyptus pellita (1); Phaius tankervilleae (1); Eucalyptus camaldulensis (1); Ocimum tenuiflorum (1); Pinus massoniana (1); Tracheophyta (1); Fraxinus excelsior (1); Fritillaria (1); Brassica napus (1); Rhipidocladum racemiflorum (1); Enhalus acoroides (1);*

UNITE Species Hypothesis	Note	SH codes	% Matched	Dectected location	Plant host/Interacting taxa
					EcM lineage<br>*/non-ectomycorrhizal (300);*
Cyphellophora vermispora	Synonym with *Cyphellophora laciniata*	SH1228267.08FU	99	America<br>Antarctica<br>Asia<br>Europe	*Canis lupus (5); Homo sapiens (4); Triticum aestivum (1); Hordeum vulgare (1);*
Exophiala alcalophila	Correct	SH1160238.08FU	100	Asia<br>America<br>Europe	***EcM lineage***<br>*/non-ectomycorrhizal (6);*
Exophiala equina	Correct	SH1185366.08FU	98	Asia<br>America<br>Europe	*Rosa rugosa (1); Corylus avellana (1); Pinus mugo (1); Betula pendula (1); Cephalanthera rubra (1); Cephalanthera damasonium (1); Microthlaspi perfoliatum (1);*<br>***EcM lineage***<br>*/non-ectomycorrhizal (1);*
Exophiala eucalyptorum	Correct	SH1231660.08FU	99	Australia<br>Europe	-
Exophiala sideris	Correct	SH1160229.08FU	100	Africa<br>America<br>Asia<br>Europe	*Sorbus aucuparia (9); Ficus benjamina (2); Juniperus deppeana (2); Phaenocoma prolifera (1);*<br>***EcM lineage***<br>*/non-ectomycorrhizal (41);*
Exophiala xenobiotica	Correct	SH1192659.08FU	100	Worldwide	*Homo sapiens (4); Ficus benjamina (3); Lophelia pertusa (2); Tracheophyta (1); Nasutitermes corniger (1); Nothofagus cunninghamii (1);*<br>***EcM lineage***<br>*/non-ectomycorrhizal (134);*
Acremonium rutilum	Correct	SH1190973.08FU	100	America<br>Europe	-
Alternaria alternata	Synonym UNITE = *Alternaria planifunda*	SH1157990.08FU	100	Africa<br>America<br>Antarctica<br>Asia<br>Europe	*Zea mays (108); Vitis vinifera (9); Ziziphus jujuba (5); Luehea seemannii (4); Malus pumila (3); Caulerpa racemosa (3); Yucca glauca (2); Hevea brasiliensis (2); Ocimum tenuiflorum (2); Ginkgo biloba (2); Artemisia capillaris (2); Miconia argentea (1); Aegiceras corniculatum (1); Brassica napus (1); Prunella vulgaris (1); Malus domestica (1); Oryza sativa (1); Acer ginnala (1); Hippophae rhamnoides (1); Plantae (1); Fraxinus excelsior (1); Tracheophyta (1); Rhododendron argyrophyllum (1); Lepidium draba (1); Pinus mugo (1); Eichhornia crassipes (1); Cupressus arizonica (1); Grevillea robusta (1); Oryza granulata (1); Quercus petraea (1); Picea (1); Taxus globosa (1); Alnus incana (1); Cecropia insignis (1);*<br>***EcM lineage***<br>*/non-ectomycorrhizal (300);*
Alternaria hordeicola	Synonym UNITE = *Alternaria metachromatica*	SH1142798.08FU	100	Africa<br>America<br>Asia<br>Australia<br>Europe	*Microthlaspi perfoliatum (19); Homo sapiens (9); Zea mays (8); Populus nigra (7); Humulus lupulus (7); Populus (6); Triticum aestivum (5); Nassella pulchra (5); Bromus diandrus (4); Malus domestica (4); Populus euphratica (3); Zea (3); Triticum (3); Dactylis glomerata (3); Tracheophyta (2); Vitis (2); Avena sativa (2); Viscum album subsp. austriacum (2); Hordeum (2); Bromus hordeaceus (2); Phalaris aquatica (1); Pinus sylvestris (1); Citrus sinensis (1); Fraxinus mandshurica (1); Fragaria vesca (1); Fraxinus ornus (1); Quercus brantii (1); Quercus ilex (1); Fraxinus pennsylvanica (1); Carex secalina (1); Crataegus pentagyna (1); Ficus religiosa (1); Hilaria jamesii (1); Fraxinus chinensis subsp. rhynchophylla (1); Fraxinus angustifolia (1); Achillea millefolium (1); Sycon ciliatum (1); Avena barbata (1); Abies alba (1); Alnus incana (1); Pinus nigra (1); Calluna vulgaris (1); Picea abies (1); Viburnum (1); Fagus sylvatica (1); Trifolium striatum (1); Medicago lupulina (1); Helianthus annuus (1); Morus alba (1); Diabrotica virgifera (1); Festuca arundinacea (1); Fagus (1); Elymus mollis (1); Brassica napus (1); Phleum pratense (1); Tethya aurantium (1); Vitis vinifera (1); Lactuca sativa (1); Capsicum annuum (1); Calamagrostis (1); Brassica*

UNITE Species Hypothesis	Note	SH codes	% Matched	Dectected location	Plant host/Interacting taxa
					nigra (1); Ammophila arenaria (1); Arabidopsis thaliana (1);
Alternaria metachromatica	Correct	SH1142798.08FU	99	Africa America Asia Australia Europe	*Microthlaspi perfoliatum (19); Homo sapiens (9); Zea mays (8); Populus nigra (7); Humulus lupulus (7); Populus (6); Triticum aestivum (5); Nassella pulchra (5); Bromus diandrus (4); Malus domestica (4); Populus euphratica (3); Zea (3); Triticum (3); Dactylis glomerata (3); Tracheophyta (2); Vitis (2); Avena sativa (2); Viscum album subsp. austriacum (2); Hordeum (2); Bromus hordeaceus (2); Phalaris aquatica (1); Pinus sylvestris (1); Citrus sinensis (1); Fraxinus mandshurica (1); Fragaria vesca (1); Fraxinus ornus (1); Quercus brantii (1); Quercus ilex (1); Fraxinus pennsylvanica (1); Carex secalina (1); Crataegus pentagyna (1); Ficus religiosa (1); Hilaria jamesii (1); Fraxinus chinensis subsp. rhynchophylla (1); Fraxinus angustifolia (1); Achillea millefolium (1); Sycon ciliatum (1); Avena barbata (1); Abies alba (1); Alnus incana (1); Pinus nigra (1); Calluna vulgaris (1); Picea abies (1); Viburnum (1); Fagus sylvatica (1); Trifolium striatum (1); Medicago lupulina (1); Helianthus annuus (1); Morus alba (1); Diabrotica virgifera (1); Festuca arundinacea (1); Fagus (1); Elymus mollis (1); Brassica napus (1); Phleum pratense (1); Tethya aurantium (1); Vitis vinifera (1); Lactuca sativa (1); Capsicum annuum (1); Calamagrostis (1); Brassica nigra (1); Ammophila arenaria (1); Arabidopsis thaliana (1);*
Ascochyta rabiei	Not in taxonomy, UNITE = *Didymella exigua*	SH1174007.08FU	97	Worldwide	*Zea mays (35); Malus pumila (9); Humulus lupulus (7); Fraxinus excelsior (6); Fagus (6); Pinus sylvestris (5); Alnus glutinosa (4); Panicum virgatum (4); Phragmites australis (4); Picea abies (3); Vitis vinifera (3); Calluna vulgaris (3); Populus (2); Sorghum bicolor (2); Buxus sempervirens (2); Populus nigra (2); Taxus globosa (2); Homo sapiens (2); Populus tremula (2); Pinus radiata (2); Phleum pratense (1); Salix cinerea (1); Taxus chinensis var. mairei (1); Buxus sempervirens var. suffruticosa (1); Puccinellia distans (1); Fagus sylvatica (1); Tillandsia (1); Hevea (1); Arabidopsis thaliana (1); Pelargonium zonale (1); Alnus incana (1); Miscanthus giganteus (1); Astacus astacus (1); Brassica napus (1); Cicer arietinum (1); Populus euphratica (1); Salix (1); Vitis (1); Phalaris arundinacea (1); Angiospermae (1); Oryza sativa (1); Pinus nigra (1); Huperzia selago (1); Protea magnifica (1); Microthlaspi perfoliatum (1);* ***EcM lineage*** */non-ectomycorrhizal (300);*
Boeremia exigua	Correct, threshold = 1%	SH1712308.08FU	100	Africa America Asia Australia Europe	*Mentha piperita (11); Solanum tuberosum (7); Microthlaspi perfoliatum (7); Tillandsia (6); Convolvulus arvensis (5); Fraxinus excelsior (4); Plantago major (4); Sorbus intermedia (3); Olea europaea (3); Linum usitatissimum (3); Glycine max (3); Picea abies (3); Cinchona pubescens (2); Vicia faba (2); Malus domestica (2); Calystegia sepium (2); Ranunculus acris (2); Ambrosia artemisiifolia (2); Ipomoea purpurea (1); Alnus incana (1); Hydrangea paniculata (1); Paeonia (1); Brassica napus (1); Salix repens (1); Glycyrrhiza glabra (1); Cycas circinalis (1); Sophora microphylla (1); Sida hermaphrodita (1); Pleurothallis coriacardia (1); Hypericum kalmianum (1); Betula pendula (1); Dactylis glomerata (1); Sambucus nigra (1); Zantedeschia aethiopica (1); Juglans hindsii (1); Fraxinus ornus (1); Heracleum sosnowskyi (1); Phaseolus vulgaris (1); Cirsium arvense (1); Phlomis younghusbandii (1); Althaea rosea (1); Althaea officinalis (1); Agapanthus (1); Humulus lupulus (1); Acroptilon repens (1); Centaurea solstitialis (1); Rudbeckia fulgida (1); Calluna vulgaris (1); Salix (1); Tanacetum cinerariifolium (1); Trifolium*

UNITE Species Hypothesis	Note	SH codes	% Matched	Dectected location	Plant host/Interacting taxa
					subterraneum (1); Nerium oleander (1); Taraxacum officinale (1); Vitis vinifera (1); Arabidopsis thaliana (1); Buxus sempervirens var. suffruticosa (1); Lavatera thuringiaca (1); Populus trichocarpa (1); Verbascum lychnitis (1); Actinidia deliciosa (1); Ipomoea batatas (1); Salvia greggii (1); Gossypium hirsutum (1); Cyclachaena xanthifolia (1); Physalis peruviana (1); ***EcM lineage*** */non-ectomycorrhizal (176);*
Botrytis caroliniana	Correct	SH1189120.08FU	100	Africa<br>America<br>Asia<br>Australia<br>Europe	*Vitis vinifera (7); Malus pumila (4); Pelargonium zonale (4); Chrysanthemum (4); Tillandsia (4); Malus domestica (3); Syringa reticulata subsp. amurensis (2); Paeonia suffruticosa (2); Vitis (2); Alnus incana (1); Eucalyptus dunnii (1); Lagerstroemia indica (1); Brassica juncea (1); Apium graveolens (1); Physalis peruviana (1); Paeonia lactiflora (1); Asclepias curassavica (1); Miconia albicans (1); Lilium (1); Abies alba (1); Peperomia ferreyrae (1); Forsythia suspensa (1); Scutellaria baicalensis (1); Codonopsis pilosula (1); Panax ginseng (1); Calendula officinalis (1); Vaccinium corymbosum (1); Angelica sinensis (1); Pinus radiata (1); Brassica napus (1); Stevia rebaudiana (1); Espeletia (1); Rosa (1); Humulus lupulus (1); Eucalyptus grandis (1); Homo sapiens (1); Populus tremula (1); Populus euphratica (1); Tethya aurantium (1); Rubus idaeus (1); Euphorbiaceae (1); Solanum lycopersicum (1); Hypericum perforatum (1); Hylurgus ligniperda (1); Linum usitatissimum (1); Phaseolus vulgaris (1); Catharanthus roseus (1); Beta vulgaris (1); Lilium longiflorum (1);* *EcM lineage* */non-ectomycorrhizal (282);*
Catenulostroma hermanusense	Correct	SH1154544.08FU	99	Africa<br>America<br>Asia<br>Australia<br>Europe	*Leucadendron tinctum (2); Hakea sericea (1); Phaenocoma prolifera (1); Pinus radiata (1);*
Cercospora sojina	Not in taxonomy, UNITE = *Sphaerulina tirolensis*	SH1194042.08FU	100	Africa<br>America<br>Asia<br>Australia<br>Europe	*Rosaceae (49); Glycine max (8); Beta vulgaris (6); Coffea arabica (5); Tectona grandis (3); Lythrum salicaria (3); Phaseolus vulgaris (3); Ensete ventricosum (3); Trifolium subterraneum (2); Armoracia rusticana (2); Zea mays (2); Chrysanthemoides monilifera (2); Acacia (2); Nicotiana tabacum (2); Eichhornia crassipes (2); Vitis (2); Pinus taeda (1); Vitis vinifera (1); Zinnia elegans (1); Phaseolus lunatus (1); Sinapis arvensis (1); Eichhornia azurea (1); Apium graveolens (1); Lygodium volubile (1); Citrus limon (1); Macrothelypteris torresiana (1); Selaginella arenicola (1); Syzygium cumini (1); Fallopia convolvulus (1); Physalis ixocarpa (1); Ocimum basilicum (1); Bletilla ochracea (1); Hydrangea macrophylla (1); Vitex (1); Taraxacum (1); Coffea (1); Glycine soja (1); Citrus sinensis (1); Impatiens balsamina (1); Molucella (1); Quercus (1); Dioscorea cayennensis (1);*
Cladosporium herbarum	Synonym: *Mycosphaerella tassiana*	SH1190878.08FU	100	Worldwide	*Zea mays (20); Juniperus deppeana (10); Coffea arabica (6); Picea abies (6); Hevea brasiliensis (4); Phragmites australis (4); Vitis (4); Cecropia insignis (3); Acropora formosa (2); Rhododendron fortunei (2); Quercus (2); Pinus monticola (2); Yucca glauca (2); Carica papaya (2); Vitis vinifera (2); Helianthus annuus (1); Epacris*

UNITE Species Hypothesis	Note	SH codes	% Matched	Dectected location	Plant host/Interacting taxa
					microphylla (1); Glycine max (1); Fagus sylvatica (1); Eucalyptus grandis (1); Saccharum officinarum (1); Poaceae (1); Ostrinia nubilalis (1); Rosa (1); Quercus nigra (1); Trifolium repens (1); Medicago polymorpha (1); Angiospermae (1); Platycladus orientalis (1); Pyrus communis (1); Calluna vulgaris (1); Ginkgo biloba (1); Silene maritima (1); Pinus ponderosa (1); Taxus globosa (1); Nothofagus cunninghamii (1); Quercus petraea (1); Sorghum vulgare (1); Grevillea robusta (1); Juniperus virginiana (1); Cucumis sativus (1); Paphiopedilum bellatulum (1); Cortaderia (1); Eucalyptus pellita (1); Phaius tankervilleae (1); Eucalyptus camaldulensis (1); Ocimum tenuiflorum (1); Pinus massoniana (1); Tracheophyta (1); Fraxinus excelsior (1); Fritillaria (1); Brassica napus (1); Rhipidocladum racemiflorum (1); Enhalus acoroides (1);<br>***EcM lineage***<br>*/non-ectomycorrhizal (300);*
Clonostachys rosea	Correct	SH1155535.08FU	100	Worldwide	*Cicer arietinum (5); Microthlaspi perfoliatum (5); Nitraria tangutorum (5); Populus trichocarpa (4); Vitis (4); Vitis vinifera (3); Brassica napus (3); Triticum aestivum (3); Pinus sylvestris (3); Rhododendron decorum (3); Pinus nigra (3); Prunus armeniaca (2); Arrhenatherum elatius (2); Quercus ilex (2); Cinnamomum verum (2); Tethya aurantium (2); Dendroctonus rufipennis (2); Chamaecrista fasciculata (1); Changnienia amoena (1); Plantae (1); Oryza granulata (1); Fritillaria unibracteata (1); Hevea brasiliensis (1); Fraxinus angustifolia (1); Saccharum officinarum (1); Arabis alpina (1); Welwitschia mirabilis (1); Capsella bursa-pastoris (1); Tetraberlinia bifoliolata (1); Gastrodia sesamoides (1); Tenebrio (Tenebrio) molitor (1); Aconitum carmichaeli (1); Lupinus albus (1); Alnus glutinosa (1); Phragmites australis (1); Epipactis albensis (1); Salix (1); Macleaya cordata (1); Metrosideros excelsa (1); Buxus sempervirens (1); Fraxinus excelsior (1); Ferula (1); Pseudotsuga menziesii var. glauca (1); Ziziphus jujuba (1); Pinus pinea (1); Lycopersicon esculentum (1); Pisum sativum (1); Zanthoxylum bungeanum (1); Anacardium excelsum (1); Vicia faba*<br>*EcM lineage*<br>*/non-ectomycorrhizal (207);*
Curvularia lunata	Correct	SH1157991.08FU	100	Africa<br>America<br>Asia<br>Australia<br>Europe	*Bouteloua gracilis (19); Homo sapiens (14); Gossypium arboreum (6); Jatropha curcas (5); Oryza sativa (4); Triticum aestivum (3); Phoenix dactylifera (3); Zea mays (3); Medicago sativa (2); Schizachyrium scoparium (2); Bouteloua curtipendula (2); Poaceae (2); Nitraria sphaerocarpa (1); Gossypium hirsutum (1); Pistacia atlantica (1); Celtis australis (1); Psammochloa villosa (1); Sorghum vulgare (1); Sorghum bicolor (1); Panicum virgatum (1); Sorghum halepense (1); Cucurbita maxima (1); Platycladus orientalis (1); Zingiber officinale (1); Microthlaspi perfoliatum (1); Nicotiana tabacum (1); Equisetum arvense (1); Platanus orientalis (1); Cymbopogon citratus (1);*<br>***EcM lineage***<br>*/non-ectomycorrhizal (184);*
Cyphellophora sessilis	Correct	SH1228270.08FU	100	America<br>Asia<br>Europe	*Pyrus communis (1); Fraxinus excelsior (1); Fiorinia externa (1);*
Devriesia pseudoamericana	Correct	SH1231799.08FU	100	America<br>Asia<br>Europe	*Picea abies (3); Malus domestica (1); Pinus koraiensis (1); Fagus sylvatica (1); Ulmus americana (1); Tilia amurensis (1);*
Didymella arachidicola	Correct	SH2232037.08FU	99	Africa<br>America<br>Asia	*Pisum sativum (30); Glycine max (4); Trifolium pratense (1); Hordeum vulgare (1); Phaseolus vulgaris (1); Petroselinum crispum (1); Holcus lanatus (1); Corchorus*

UNITE Species Hypothesis	Note	SH codes	% Matched	Dectected location	Plant host/Interacting taxa
				Australia<br>Europe	*olitorius (1); Cirsium arvense (1); Sophora microphylla (1); Disporopsis fuscopicta (1); Sambucus pubescens (1); Liriodendron tulipifera (1); Trifolium alexandrinum (1);*<br>***EcM lineage***<br>*/non-ectomycorrhizal (94);*
Drechslera phlei	Synonym, Pleosporaceae (*Drechslera phlei*)	SH1192926.08FU	100	Europe	-
Edenia gomezpompae	Synonyme, Pezizomycotina (*Setophoma terrestris*)	SH1146626.08FU	100	Africa<br>America<br>Asia<br>Australia<br>Europe	*Poaceae (47); Nicotiana occidentalis (11); Nicotiana benthamiana (9); Bouteloua gracilis (9); Zea mays (6); Capsicum annuum (4); Brachiaria (4); Nicotiana simulans (4); Allium fistulosum (3); Erioscirpus comosus (3); Triticum aestivum (3); Vanilla planifolia (3); Allium cepa (3); Paeonia lactiflora (3); Medicago minima (2); Fagopyrum esculentum (2); Ephedra distachya (2); Cucurbita maxima (2); Dioscorea zingiberensis (1); Fagopyrum tataricum (1); Phyllostachys viridiglaucescens (1); Vitis vinifera (1); Disporopsis fuscopicta (1); Miscanthus giganteus (1); Glycine max (1); Tectona grandis (1); Andropogon gerardi (1); Solanum tuberosum (1); Mimosa annularis (1); Thlaspi perfoliatum (1); Ailanthus altissima (1); Malus pumila (1); Lycium barbarum (1); Orchis provincialis (1); Salix (1); Harrisia eriophora (1); Coffea arabica (1); Dittrichia viscosa (1); Holcus lanatus (1); Achyranthes aspera (1); Annona spraguei (1); Nephrolepis brownii (1); Phaseolus vulgaris (1); Armoracia rusticana (1); Populus trichocarpa (1); Gloriosa superba (1);*
Erysiphe polygoni	Correct, threshold = 1%	SH1732211.08FU	100	America<br>AntarcticaAsia<br>Europe	*Polygonum aviculare (5); Rumex obtusifolius (3); Polygonum erectum (1); Polygonum arenastrum (1); Antigonon leptopus (1); Rumex crispus (1); Rheum rhabarbarum (1); Fagopyrum esculentum (1);*
Fusarium domesticum	UNITE = Nectriaceae (*Fusarium*)	SH1173621.08FU	100	America<br>Asia<br>Europe	*Brassica napus (3); Carica papaya (1);*
Fusarium fujikuroi	Synonym, *Fusarium proliferatum*	SH1212031.08FU	100	Africa<br>America<br>Asia<br>Australia<br>Europe	*Brachiaria (13); Brassica tournefortii (12); Zea mays (8); Oryza sativa (6); Cucumis sativus (3); Stachys affinis (3); Capsella bursa-pastoris (2); Solanum lycopersicum (2); Leucas aspera (1); Cucumis melo (1); Hypericum perforatum (1); Azadirachta indica (1); Rhizophora mangle (1); Bouteloua dactyloides (1); Commelina erecta (1); Cajanus cajan (1); Mentha x piperita (1); Triticum aestivum (1); Toxicodendron sylvestre (1); Tabernaemontana alternifolia (1); Dendrobium moniliforme (1); Jasminum sambac (1); Fragaria chiloensis (1); Phaseolus vulgaris (1); Iris sibirica (1); Aquilaria malaccensis (1); Sauropus androgynus (1);*
Fusarium oxysporum	Synonym, *Gibberella fujikuroi (Fusarium oxysporum)*	SH1212030.08FU	100	Africa<br>America<br>Asia<br>Australia<br>Europe	*Cicer arietinum (25); Phaseolus (13); Homo sapiens (11); Dracaena (7); Cecropia insignis (7); Ricinus communis (6); Capsicum annuum (5); Warburgia ugandensis (4); Malus pumila (4); Heterodera schachtii (3); Dittrichia viscosa (3); Poaceae (2); Cymbidium (2); Gossypium hirsutum (2); Vicia faba (2); Bouteloua gracilis (2); Coffea (2); Cucurbitaceae (2); Vitis vinifera (2); Lycopersicon esculentum (2); Pinus sylvestris (2); Cephalanthera damasonium (1); Sporobolus cryptandrus (1); Grevillea robusta (1); Phaseolus vulgaris (1); Pecteilis susannae (1); Dactylis glomerata (1); Holcus lanatus (1); Ammophila arenaria (1); Ginkgo biloba (1); Zea mays (1); Coffea arabica (1); Hippophae rhamnoides (1); Phascolarctos cinereus (1); Artemisia annua (1); Yucca glauca (1); Solanum tuberosum (1); Salix (1);*<br>*EcM lineage*<br>*/non-ectomycorrhizal (46);*
Fusarium poae	Synonym, *Fusarium*	SH1212037.08FU	100	Africa<br>America<br>Asia	*Humulus lupulus (5); Solanum tuberosum (4); Zea mays (3); Poaceae (3); Posidonia oceanica (2); Fraxinus excelsior (2); Malus domestica (2); Phaseolus vulgaris*

UNITE Species Hypothesis	Note	SH codes	% Matched	Dectected location	Plant host/Interacting taxa
	venenatum (*Fusarium poae*)			Australia<br>Europe	*(2); Dactylis glomerata (1); Iris sibirica (1); Elaeis guineensis (1); Taxus globosa (1); Capsella bursa-pastoris (1); Papaver somniferum (1); Vitis vinifera (1); Aconitum vilmorinianum (1); Physalis peruviana (1);*
Fusarium proliferatum	Correct	SH1212031.08FU	100	Africa<br>America<br>Asia<br>Australia<br>Europe	*Brachiaria (13); Brassica tournefortii (12); Zea mays (8); Oryza sativa (6); Cucumis sativus (3); Stachys affinis (3); Capsella bursa-pastoris (2); Solanum lycopersicum (2); Leucas aspera (1); Cucumis melo (1); Hypericum perforatum (1); Azadirachta indica (1); Rhizophora mangle (1); Bouteloua dactyloides (1); Commelina erecta (1); Cajanus cajan (1); Mentha x piperita (1); Triticum aestivum (1); Toxicodendron sylvestre (1); Tabernaemontana alternifolia (1); Dendrobium moniliforme (1); Jasminum sambac (1); Fragaria chiloensis (1); Phaseolus vulgaris (1); Iris sibirica (1); Aquilaria malaccensis (1); Sauropus androgynus (1);*
Fusarium solani	Synonym, *Fusarium pseudensiforme (Fusarium solani)*	SH1173587.08FU	100	Africa<br>America<br>Asia<br>Australia<br>Europe	*Homo sapiens (25); Pisum sativum (12); Phaseolus (11); Dittrichia viscosa (4); Cecropia insignis (3); Chelonia mydas (3); Yucca glauca (2); Morus (2); Cicer arietinum (2); Coffea (2); Caretta caretta (2); Poaceae (2); Ailanthus altissima (2); Arachis hypogaea (2); Fabaceae (2); Elaeis guineensis (2); Withania somnifera (1); Hevea (1); Eucommia ulmoides (1); Populus alba (1); Medicago minima (1); Pinus taeda (1); Plantae (1); Chrysanthemum x morifolium (1); Vitex (1); Citrullus lanatus (1); Panax (1); Cymbidium (1); Vitis vinifera (1); Lycopersicon esculentum (1); Citrullus vulgaris (1); Dioscorea zingiberensis (1); Pinus massoniana (1); Phalaenopsis (1); Solanum tuberosum*<br>*EcM lineage*<br>*/non-ectomycorrhizal (299);*
Gaeumannomyces radicicola	Correct	SH1211553.08FU	100	Africa<br>America<br>Asia<br>Australia<br>Europe	*Plantae (11); Stenotaphrum secundatum (11); Triticum aestivum (8); Zea mays (4); Ammophila breviligulata (2); Amomum siamense (1); Chrysopogon zizanioides (1); Alpinia malaccensis (1); Brachiaria (1); Pennisetum clandestinum (1); Oryza sativa (1); Tridens flavus (1); Zingiber mioga (1); Oryza granulata (1); Agrostis stolonifera (1); Ammophila arenaria (1); Avena sativa (1); Hordeum vulgare (1); Holcus lanatus (1); Zingiber officinale (1);*
Gibberella tricincta	Correct	SH1173588.08FU	100	Africa<br>America<br>Asia<br>Australia<br>Europe	*Microthlaspi perfoliatum (52); Brassica (12); Ipomoea batatas (6); Cicer arietinum (5); Pinus halepensis (4); Ferula (4); Sclerotinia sclerotiorum (4); Buxus sempervirens (3); Gaultheria poeppigii (2); Ailanthus altissima (2); Platanus orientalis (2); Triticum aestivum (2); Paeonia suffruticosa (2); Salix (2); Tethya aurantium (2); Vitis vinifera (2); Brassica napus (2); Ventenata dubia (2); Linum usitatissimum (1); Pinus nigra (1); Platycladus orientalis (1); Medicago sativa (1); Valeriana officinalis (1); Pseudotsuga menziesii var. glauca (1); Carthamus tinctorius (1); Impatiens balsamina (1); Cistus creticus (1); Lespedeza davurica (1); Aronia melanocarpa (1); Pinus sylvestris (1); Panax (1); Ambrosia artemisiifolia (1); Artemia franciscana (1); Pisum (1); Malus pumila (1); Picea abies (1); Glycine max (1); Poaceae (1); Bouteloua gracilis (1); Fritillaria unibracteata (1); Actinidia arguta (1); Allium cepa (1); Yucca glauca (1); Eucommia ulmoides (1); Homo sapiens (1); Beta vulgaris (1); Panax notoginseng (1); Geum macrophyllum (1);*<br>***EcM lineage***<br>*/non-ectomycorrhizal (291);*
Gibberella zeae	Correct	SH1212032.08FU	97	Africa<br>America<br>Asia<br>Australia<br>Europe	*Zea mays (12); Triticum aestivum (9); Homo sapiens (5); Triticum (2); Oryza sativa (2); Solanum tuberosum (2); Piptadenia adiantoides (2); Vitis vinifera (1); Begonia sodiroi (1); Piper arboreum (1); Bauhinia brachycalyx (1); Ipomoea batatas (1); Saponaria officinalis (1);*

UNITE Species Hypothesis	Note	SH codes	% Matched	Dectected location	Plant host/Interacting taxa
					Microthlaspi perfoliatum (1); Pacifastacus leniusculus (1); Carya illinoinensis (1); Malus pumila (1); Brassica (1); Sorghum bicolor (1); Camellia sinensis (1); Diabrotica virgifera subsp. virgifera (1); Miscanthus giganteus (1); Grevillea robusta (1); Pinus sylvestris (1); Ostrinia nubilalis (1); Dactylis glomerata (1); Glycine max (1); Orthotomicus erosus (1); Coffea arabica (1); Lepidoptera (1);
Gibellulopsis nigrescens	Synonym, threshold = 2%, *Acremonium stromaticum (Gibellulopsis nigrescens)*	SH1487539.08FU	100	America Asia Europe	-
Golovinomyces montagnei	Correct	SH1210316.08FU	99	Asia Europe	*Capsicum annuum (3);*
Golovinomyces riedlianus	Correct	SH1210318.08FU	99	Asia Europe	*Galium verum (1); Galium album (1);*
Knufia cryptophialidica	Synonym, *Knufia endospore (Knufia cryptophialidica)*	SH1180804.08FU	100	Europe	*Corylus americana (2); Crataegus punctata (1); Salix alba (1); Ulmus laevis (1); Salix alba x Salix fragilis (1); Tilia cordata (1); Thuja occidentalis (1);* ***EcM lineage*** */non-ectomycorrhizal (7);*
Leptosphaeria rubefaciens	Correct	SH1227794.08FU	100	Europe	*Fraxinus lanuginosa (1); Pinus cembra (1); Fraxinus excelsior (1); Acer mandshuricum (1); Salix alba (1); Salix alba x Salix fragilis (1); Corylus avellana (1); Liriodendron tulipifera (1);*
Monographella nivalis	Correct	SH1179475.08FU	100	America AntarcticaAsia Australia Europe	*Festuca rubra (4); Lolium perenne (4); Triticum aestivum (3); Agrostis palustris (2); Ammophila arenaria (2); Plantae (1); Festulolium (1); Quercus macrocarpa (1); Corylus avellana (1); Poa pratensis (1); Homo sapiens (1); Holcus lanatus (1); Agrostis stolonifera (1); Neottia ovata (1); Carpinus cordata (1); Secale cereale (1); Picea pungens (1); Lolium multiflorum (1); Pinus sylvestris (1); Nothofagus cunninghamii (1);*
Nectria ramulariae	Correct	SH1173590.08FU	100	America Asia Europe	*Fagus sylvatica (27); Microthlaspi perfoliatum (6); Picea abies (3); Betula pendula (2); Arabis alpina (2); Malus sylvestris (2); Alnus incana (1); Pinus sylvestris (1); Pseudotsuga menziesii (1); Phragmites australis (1); Pinus cembra (1); Abies holophylla (1); Cephalanthera longibracteata (1); Brassica napus (1); Andropogon gerardii (1); Beta vulgaris (1); Vitis (1); Abies alba (1);*
Neoascochyta graminicola	Correct	SH1920701.08FU	100	America Asia Australia Europe	*Hordeum vulgare (2); Fraxinus excelsior (1); Triticum aestivum (1); Humulus lupulus (1); Festuca paniculata (1); Ulmus laevis (1); Liriodendron tulipifera (1);*
Neocatenulostroma abietis	Correct	SH2316213.08FU	100	Europe	*Juniperus communis (1); Abies (1); Pinus sylvestris (1); Larix sibirica (1);*
Neodevriesia capensis	Correct	SH1175695.08FU	98	AntarcticaEurope	*Proteaceae (1); Pinus radiata (1); Protea repens (1);*
Oculimacula yallundae	Correct	SH1405820.08FU	99	America Africa Europe	*Triticum aestivum (4); Brassica napus (1); Calamagrostis purpurea (1);*
Paraphoma chrysanthemicola	*Paraphoma rhaphiolepidis (Paraphoma chrysanthemicola)*	SH1193180.08FU	100	America Asia Australia Europe	*Microthlaspi perfoliatum (89); Thlaspi perfoliatum (7); Arrhenatherum elatius (5); Phlomis younghusbandii (4); Ficus (4); Capsella bursa-pastoris (3); Fagus sylvatica (3); Brassica napus (2); Chrysanthemum x morifolium (2); Lomariopsis vestita (1); Campyloneurum serpentinum (1); Picea abies (1); Tectaria incisa (1); Pinus ponderosa (1); Hedysarum scoparium (1); Phalaris arundinacea (1); Pinus sylvestris (1); Bupleurum chinense (1); Gaultheria poeppigii (1); Vincetoxicum rossicum (1); Lespedeza davurica (1); Platanus orientalis (1); Cichorium intybus (1); Acer platanoides (1); Pinus cembra (1); Cypripedium macranthos (1); Fraxinus excelsior (1); Achillea millefolium (1); Populus trichocarpa (1); Fragaria vesca*

UNITE Species Hypothesis	Note	SH codes	% Matched	Dectected location	Plant host/Interacting taxa
					(1); Zanthoxylum bungeanum (1); Ambrosia artemisiifolia (1); Vitis vinifera (1); Glycine soja (1);
Plectosphaerella cucumerina	*Acremonium nepalense (Plectosphaerella cucumerina)*	SH1234026.08FU	100	America Africa Asia Australia Europe	*Microthlaspi perfoliatum (20); Nicotiana occidentalis (7); Arabidopsis thaliana (3); Brassica napus (3); Dittrichia viscosa (2); Capsella bursa-pastoris (2); Alnus incana (1); Nicotiana benthamiana (1); Betula pendula (1); Ranunculus acris (1); Piper tiliifolium (1); Eucalyptus gunnii (1); Berberis thunbergii (1); Dendrobium catenatum (1); Cichorium intybus (1); Capsicum annuum (1); Eleocharis erythropoda (1); Siparuna aspera (1); Piper andreanum (1); Carex bigelowii (1); Ammophila arenaria (1); Fraxinus excelsior (1); Ambrosia artemisiifolia (1); Panax notoginseng (1); Homo sapiens (1); Solidago canadensis (1); Epipactis helleborine (1); Plantae (1); Elymus farctus (1); Asclepias syriaca (1);*
Protomyces inouyei	Correct	SH1235961.08FU	100	America Europe	-
Pyrenophora lolii	Correct	SH1431037.08FU	100	America Europe	*Nassella pulchra (6); Bromus diandrus (6); Avena barbata (5); Elymus glaucus (2); Lolium perenne (1); Lolium multiflorum (1); Wheat*
Pyrenophora teres	*Pyrenophora tritici-repentis*	SH1192922.08FU	100	America Asia Europe	*Triticum (58); Hordeum vulgare (21); Elymus glaucus (15); Triticum durum (10); Bromus inermis (5); Thinopyrum intermedium (4); Lolium multiflorum (3); Hordeum (2); Zea mays (2); Leymus cinereus (1); Fraxinus excelsior (1); Stipa grandis (1); Hesperostipa comata (1); Elymus angustus (1); Lythrum salicaria (1); Elymus repens (1); Bromus tectorum (1); Poa pratensis (1); Acer pseudosieboldianum (1);*
Pyrenophora tritici-repentis	Correct	SH1192922.08FU	100	America Asia Europe	*Triticum (58); Hordeum vulgare (21); Elymus glaucus (15); Triticum durum (10); Bromus inermis (5); Thinopyrum intermedium (4); Lolium multiflorum (3); Hordeum (2); Zea mays (2); Leymus cinereus (1); Fraxinus excelsior (1); Stipa grandis (1); Hesperostipa comata (1); Elymus angustus (1); Lythrum salicaria (1); Elymus repens (1); Bromus tectorum (1); Poa pratensis (1); Acer pseudosieboldianum (1);*
Ramularia collo-cygni	Correct	SH1577465.08FU	99	America Africa Australia Europe	*Nassella pulchra (27); Hordeum vulgare (11); Bromus diandrus (4); Lolium perenne (4); Bromus hordeaceus (2); Acer mandshuricum (1); Secale cereale (1); Calluna vulgaris (1); Corylus avellana (1); Salix alba x Salix fragilis (1); Pyrus communis (1);*
Rhexocercosporidium panacis	Not in taxonomy, UNITE = *Rhynchosporium commune*	SH1173280.08FU	99	Worldwide	*Microthlaspi perfoliatum (20); Juniperus communis (10); Vitis vinifera (7); Actinidia (6); Fragaria vesca (5); Actinidia deliciosa (4); Picea glauca (3); Vitis (2); Carex sprengelii (2); Malus domestica (2); Fraxinus excelsior (2); Populus tremula (2); Tracheophyta (2); Elymus farctus (2); Lythrum salicaria (2); Picea abies (2); Persicaria amphibia (2); Ficus religiosa (1); Rosa rugosa (1); Brassica napus (1); Cyclopterus lumpus (1); Orchis militaris (1); Cypripedium californicum (1); Sycon ciliatum (1); Arabis alpina (1); Alnus incana (1); Citrus reticulata (1); Elymus mollis (1); Hordeum murinum (1); Populus deltoides (1); Liquidambar styraciflua (1); Angiospermae (1); Malus sylvestris (1); Pinus monticola (1); Populus alba (1);*
Septoriella hirta	Synonym, *Septoriella hubertusii*	SH1157055.08FU	100	America AntarcticaAsia Australia Europe	*Calamagrostis purpurea (11); Festuca rubra (10); Dactylis glomerata (8); Equisetum arvense (7); Phragmites australis (6); Ammophila arenaria (6); Triticum aestivum (4); Ranunculus acris (3); Zea mays (3); Arundo plinii (3); Microthlaspi perfoliatum (3); Kobresia simpliciuscula (2); Elymus mollis (2); Picea abies (2); Agropyron repens (2); Calluna vulgaris (2); Equisetum hyemale (2); Carex rostrata (2); Ulmus americana (2); Carex acutiformis (2); Polylepis pauta (1); Pinus radiata (1); Ficus religiosa (1); Quercus robur (1); Rosa canina (1); Phalaris aquatica (1); Pyrus communis (1); Stipa grandis (1); Deschampsia caespitosa*

UNITE Species Hypothesis	Note	SH codes	% Matched	Dectected location	Plant host/Interacting taxa
					(1); Panicum virgatum (1); Fraxinus chinensis subsp. rhynchophylla (1); Ammophila breviligulata (1); Calamagrostis arundinacea (1); Lycopodium annotinum (1); Abies alba (1); Agrostis stolonifera (1); Trisetum spicatum (1); Phleum pratense (1); Arrhenatherum elatius (1); Picea obovata (1); Fagus sylvatica (1); Deschampsia flexuosa (1); Angiospermae (1); Betula pendula (1); Populus tremula (1);
Setomelanomma holmii	Correct	SH1216099.08FU	100	America Asia Europe	*Picea pungens (3); Fraxinus mandshurica (1); Pinus sylvestris (1);*
Taphrina carpini	Correct	SH1153417.08FU	100	America Asia Europe	*Fagus (1); Picea abies (1); Pinus sylvestris (1); Quercus pyrenaica (1); Ulmus laevis (1); Prunus spinosa (1); Fraxinus excelsior (1); Quercus (1); Salix fragilis (1); Corylus avellana (1); Tilia cordata (1); Pyrus communis (1); Corylus americana (1); Populus tremula (1);*
Torula herbarum	Correct	SH1182954.08FU	100	Europe	*Phragmites australis (1);*
Verticillium albo-atrum	Correct	SH1154043.08FU	100	Africa America Asia Australia Europe	*Ailanthus altissima (4); Olea europaea (3); Plantae (2); Capsella bursa-pastoris (2); Vaccinium corymbosum (2); Gossypium hirsutum (2); Vicia faba (1); Abelmoschus esculentus (1); Stevia rebaudiana (1); Brassica napus (1); Solanum tuberosum (1); Capsicum annuum (1); Lycium barbarum (1); Globodera pallida (1); Helianthus annuus (1); Solanum lycopersicum (1); Solanum melongena (1); Pisum sativum (1); Prunus persica (1); Tetragonia expansa (1); Glycyrrhiza uralensis (1);*
Verticillium nonalfalfae	Synonym, *Verticillium albo-atrum (Verticillium dahliae)*	SH1154043.08FU	99	Africa America Asia Australia Europe	*Ailanthus altissima (4); Olea europaea (3); Plantae (2); Capsella bursa-pastoris (2); Vaccinium corymbosum (2); Gossypium hirsutum (2); Vicia faba (1); Abelmoschus esculentus (1); Stevia rebaudiana (1); Brassica napus (1); Solanum tuberosum (1); Capsicum annuum (1); Lycium barbarum (1); Globodera pallida (1); Helianthus annuus (1); Solanum lycopersicum (1); Solanum melongena (1); Pisum sativum (1); Prunus persica (1); Tetragonia expansa (1); Glycyrrhiza uralensis (1);*
Waitea circinata	Synonym, *Ceratobasidiaceae (Waitea circinata)*	SH1211869.08FU	100	America Asia Europe	-

* Nilsson, R. H., Larsson, K.-H., Taylor, A. F. S., Bengtsson-Palme, J., Jeppesen, T. S., Schigel, D., et al. (2019). The UNITE database for molecular identification of fungi: handling dark taxa and parallel taxonomic classifications. Nucleic Acids Res. 47, D259–D264. doi: 10.1093/nar/gky1022

Table S2. Information on relative abundance (%) of fungal pathogens detected in poly(butylene succinate-co-adipate) (PBSA).

Table S3. Information on relative abundance (%) of bacterial pathogens detected in poly(butylene succinate-co-adipate) (PBSA).

Table S4. Fungi detected in control soils of ambient (PSA1) and future putative (PSF1) climate conditions before PBSA addition. PSA2, PSA3 and PSA4 represent soils of ambient climate 30, 180 and 328 days after PBSA addition. PSF2, PSF3 and PSF4 represent soils of future putative climate 30, 180 and 328 days after PBSA addition.

Table S5. Bacteria detected in control soils of ambient (PSA1) and future putative (PSF1) climate conditions before PBSA addition. PSA2, PSA3 and PSA4 represent soils of ambient climate 30, 180 and 328 days after PBSA addition. PSF2, PSF3 and PSF4 represent soils of future putative climate 30, 180 and 328 days after PBSA addition.

Table S6. The effects of climate conditions, incubation time and their interaction on the fungal relative abundances (frequency of detection) of individual pathogens were assessed by time-series analysis (repeated ANOVA).

Table S7. The effects of climate conditions, incubation time and their interaction on the bacterial relative abundances (frequency of detection) of individual pathogens were assessed by time-series analysis (repeated ANOVA).

Table S8. Microbial taxa associated with degradation of poly(butylene succinate-co-adipate) (PBSA) and polyethylene (PE). Microbial taxa were obtained from a comprehensive plastics microbial biodegradation database published by Gan, Z., and Zhang, H. (2019)**.

**Gan, Z., and Zhang, H. (2019). PMBD: a comprehensive plastics microbial biodegradation database. Database 2019:baz119. doi: 10.1093/database/baz119
